# Ellagic Acid Alleviates Diquat-Induced Jejunum Oxidative Stress in C57BL/6 Mice through Activating Nrf2 Mediated Signaling Pathway

**DOI:** 10.3390/nu14051103

**Published:** 2022-03-05

**Authors:** Xiangyu Zhang, Shilan Wang, Yujun Wu, Xiaoyi Liu, Junjun Wang, Dandan Han

**Affiliations:** State Key Laboratory of Animal Nutrition, College of Animal Science and Technology, China Agricultural University, Beijing 100193, China; 17714365719@163.com (X.Z.); wslivy@163.com (S.W.); yujun@cau.edu.cn (Y.W.); l1ux1aoy1@163.com (X.L.); wangjj@cau.edu.cn (J.W.)

**Keywords:** ellagic acid, diquat, jejunum, oxidative stress, Nrf2

## Abstract

Ellagic acid (EA) is the main constituent found in pomegranate rind, which has anti-inflammatory and antioxidant effects. However, whether EA can alleviate diquat-induced oxidative stress is still unknown. Here, the effects and mechanisms of EA on jejunum oxidative stress induced by diquat was investigated. Oxidative stress was induced in mice by administrating diquat (25 mg/kg body weight) followed by treatment with 100 mg/kg body weight EA for 5 days. Results showed that oral administration of EA significantly ameliorated diquat-induced weight loss and oxidative stress (*p* < 0.05) evidenced by reduced ROS production in the jejunum. Furthermore, EA up-regulated the mRNA expression of the antioxidant enzymes (Nrf2, GPX1 and HO-1) when mice were challenged with diquat, compared with the diquat group (*p* < 0.05). Importantly, pharmacological inhibition of Nrf2 by ML385 counteracted the EA-mediated alleviation of jejunum oxidative stress, as evidence by body weight and ROS production. Also, immunohistochemistry staining confirmed the markedly decreased jejunal Nrf2 expression. The up-regulated effect on NQO1 and HO-1 mRNA expression induced by EA was diminished in mice treated with ML385 (*p* < 0.05). Together, our results demonstrated that therapeutic and preventative EA treatment was effective in reducing weight loss and oxidative stress induced by diquat through the Nrf2 mediated signaling pathway.

## 1. Introduction

Reactive oxygen species (ROS) accumulation and oxidative stress are important causes of many diseases [1,2]. Under normal physiological conditions, the production of oxidants and antioxidants in biological systems is balanced. When the body’s antioxidant defense system fails to eliminate excessive ROS production, tissues such as intestinal tissue will suffer from oxidative damage [3]. As the main place for nutrient digestion and absorption, the intestine is particularly important for the growth and development of the body. At the same time, because the intestine is located at the junction of the body and its lumen environment, it is more vulnerable to oxidative damage, which has adverse effects on the body [4,5,6]. Some studies have confirmed that excessive ROS accumulation in the intestine can damage the morphology of intestinal villi, increase intestinal mucosal permeability, and is associated with reduced life expectancy [7,8].

Nuclear factor erythroid 2-related factor 2 (Nrf2) is a transcription factor that protects from the generation of ROS [9]. When oxidative stress occurs, Nrf2 detaches from Kelch-like ECH-associated protein 1 (KEAP1, which binds with Cullin 3) and then enters the nucleus [10]. Furthermore, Nrf2 combines with the antioxidant response elements (AREs) and induces expression of antioxidant enzymes such as superoxide dismutase (SOD1), quinone oxidoreductase 1 (NQO1), catalase (CAT) and heme oxygenase 1 (HO-1) [11]. At the same time, the downstream GSH redox system will be activated, such as glutathione peroxidase (GPX). Finally, the oxidative stress will be inhibited [11]. 

Ellagic acid (EA) is a kind of natural phenolic compound, which is considered the active compound for the antioxidant properties of pomegranate [12]. It has high antioxidant and radical scavenging activity [13]. EA could ameliorate ROS overproduction induced by high-glucose in T2DM HepG2 cells through miR-223-mediated Nrf2 activation [14]. Additionally, its intestinal metabolites, urolithin A could protect gut barrier integrity through the Nrf2 mediated pathway [15], and urolithin B could alleviate myocardial ischemia/reperfusion injury by activating Nrf2 [16]. 

Although EA has demonstrated beneficial effects on intestinal health, information on how EA reduces intestinal oxidative damage has been limited. Therefore, this study aims to explore the therapeutic effects of EA on jejunum oxidative stress in mice and explore the mechanisms. To better determine the effects and mechanisms of EA, we utilized the ML385 (specific Nrf2 inhibitor) [17,18] to explore whether the alleviation of oxidative stress by EA was dependent upon Nrf2. 

## 2. Materials and Methods

All experiments in this study received approval by the China Agricultural University Animal Care and Use Committee (AW21102202-1-2).

### 2.1. Chemicals

Ellagic acid was purchased from Acros Organics (Belgium, WI, USA), with a purity of 97%. Diquat (average molecular weight: 344.05) was derived from Shanghai Fusheng Biotechnology Co., Ltd. (Shanghai, China). ML385 was derived from Glpbio (Montclair, CA, USA).

### 2.2. Animals and Treatment

C57BL/6J mice were obtained from SPF Biotechnology Co., Ltd. (Beijing, China) and underwent a week adaptation period before treatment. Mice were kept at 22–25 °C with a 12 h light and dark cycle. Standard diet and clean water were freely available for the mice. A previous study has shown that estrogen can regulate antioxidant capacity through Nrf2. To reduce the potential effects of estrogen, male mice were used as experimental subjects in this study [19].

Therapeutic experiment 1: Twenty-four mice were randomly assigned to four experimental groups (*n* = 6/group; body weight 15.50 ± 0.73 g): i. Control group (CON), every mouse was intraperitoneally injected with 200 μL phosphate buffer saline (PBS) on day 1, and then orally administered with 100 μL PBS every day; ii. Diquat group, every mouse was intraperitoneally injected with 25 mg/kg BW diquat dissolved in 200 μL PBS on day 1, then orally administered with 100 μL PBS every day; iii. Ellagic acid group (EA), every mouse was intraperitoneally injected with 200 μL phosphate buffer saline (PBS) on day 1, and then orally administered with 100 mg/kg BW EA every day; iv. Diquat + EA group, every mouse was intraperitoneally injected with 25 mg/kg BW diquat dissolved in 200 µL PBS on day 1, and then orally administered with 100 mg/kg BW EA every day. On day 5, all mice were sacrificed for sample collection. Two sections of tissue from the middle jejunum were stored at −80 °C in 4% paraformaldehyde solution for analysis.

Therapeutic experiment 2: Twelve mice were randomly assigned to two experimental groups (*n* = 6/group; body weight 15.50 ± 0.73 g): i. Diquat group, every mouse was intraperitoneally injected with 25 mg/kg BW diquat dissolved in 200 μL PBS on day 1, then orally administered with 100 μL PBS every day; ii. Diquat + EA group, every mouse was intraperitoneally injected with 25 mg/kg BW diquat dissolved in 200 μL PBS on day 1, and then orally administered with 100 mg/kg BW EA every day. EA (100 mg/kg BW/day) was prepared in suspension in PBS with 100 μL per mouse. (Figure S1a). The time required for each mouse to recovery to its initial body weight before diquat injection was recorded.

Therapeutic experiment 3: Twenty-four mice were randomly assigned to four experimental groups (*n* = 6/group; body weight 17.40 ± 1.10g): i. Control group (CON), every mouse was intraperitoneally injected with 200 μL phosphate buffer saline (PBS) on day 1, and then orally administered with 100 μL PBS every day; ii. Diquat group, every mouse was intraperitoneally injected with 25 mg/kg BW diquat dissolved in 200 μL PBS on day 1, then orally administered with 100 μL PBS every day; iii. Diquat + EA group, every mouse was intraperitoneally injected with 25 mg/kg BW diquat dissolved in 200 μL PBS on day 1, and then orally administered with 100 mg/kg BW EA every day. iv. Diquat + EA + ML385 group, every mouse was intraperitoneally injected with 25 mg/kg BW diquat dissolved in 200 μL PBS on day 1, then orally administered with 100 mg/kg BW EA and intraperitoneally injected with 30 mg/kg BW ML385 every day. EA (100 mg/kg BW/day) was prepared in suspension in PBS with 100 μL per mouse. ML385 (a specific Nrf2 inhibitor) was prepared in suspension in PBS with 100 μL per mouse. On day 5, all mice were sacrificed. The sample collection was the same as the therapeutic experiment 1. 

Preventive experiment: Twenty-four mice were randomly divided into four groups (*n* = 6/group; body weight 14.03 ± 2.26 g): i. Control group, every mouse was orally administered with 100 μL PBS every day, and then intraperitoneally injected with 200 μL PBS on day 14; ii. Diquat group, every mouse was orally administered with 100 μL PBS every day, and then intraperitoneally injected with 25 mg/kg BW diquat dissolved in 200 μL PBS on day 14; iii. Ellagic acid group (EA), every mouse was orally administered with 100 mg/kg BW EA every day, and then intraperitoneally injected with 200 μL PBS on day 14; iv. EA + Diquat group, every mouse was orally administered with 100 mg/kg BW EA every day, and then intraperitoneally injected with 25 mg/kg BW diquat dissolved in 200 μL PBS on day 14. EA (100 mg/kg BW/day) was prepared in suspension in PBS with 100 μL per mouse. The body weights were recorded on the 1st day, 7th day and 14th day. On the 15th day of the trial, all mice were weighed and sacrificed. The sample collection was the same as the therapeutic experiment 1. 

### 2.3. Detection of Serum Indexes

The aspartate amino-transferase (AST) and alanine aminotransferase (ALT) assay kits were procured from Weiyi Biological Technology Co., Ltd. (Hangzhou, China). The alkaline phosphatase (ALP) assay kit was procured from BioSino Bio-Technology & Science Inc (Beijing, China). The assay kits of ROS, total antioxidant capacity (T-AOC), glutathione peroxidase (GSH-Px) and malondialdehyde (MDA) were procured from Beijing Sinouk Institute of Biological Technology (Beijing, China). The levels of ALT, AST, ALP, ROS, T-AOC, GSH-Px and MDA in serum of mice were detected by the above kits according to the corresponding protocols, respectively. 

### 2.4. Detection of ROS Level in Jejunum

Frozen sections were prepared from fresh jejunum. Frozen slides were restored to room temperature and obvious liquid eliminated. The objective tissue was marked with a liquid blocker pen. Spontaneous fluorescence quenching reagent was added and the section was incubated for 5 min, and then washed in running tap water for 10 min. ROS staining solution was added to the marked area, and incubated at 37 °C for 30 min in a dark place. In a Rocker device, the section was washed three times with PBS (pH 7.4), for 5 min each. They were incubated with DAPI solution for 10 min in a dark place at room temperature. They were washed three times with PBS (pH 7.4) in a Rocker device, for 5 min each. Residual liquid was removed, then a coverslip was applied with anti-fade mounting medium. Finally, the sections were observed under a fluorescence microscope (Axio Imager.M2, Zeiss, Oberkochen, Germany). 

### 2.5. Quantification of Intestinal Antioxidant Gene Expressions of Mice

Total RNA from the jejunum tissue was extracted using the RNA pure Total RNA kit (Aidlab Biotechnologies Co., Ltd., Beijing, China) according to the protocol and reverse transcribed into cDNA using a PrimeScript™ RT reagent Kit with gDNA Eraser RR047A (Takara Bio INC., Shiga, Japan). Quantitative reverse transcription PCR (RT-qPCR) was performed using the TB Green^®^ Premix Ex Taq ™ RR420Q (Takara Bio INC., Shiga, Japan) on a Light Cycler System (Roche, South San Francisco, CA, USA). The data were analyzed following the 2^−ΔΔCt^ method and calculated using β-actin as the normalization control. Primers for RT-qPCR (Table 1) were synthesized by Xinshidai Zhonghe Technology Co., Ltd. (Beijing, China). Nrf2, GPX1, NQO-1, SOD1, HO-1, SOD2 and CAT mRNA expressions in jejunum tissue were measured.

### 2.6. Hematoxylin and Eosin Staining

Intestinal samples of mice were removed from 4% paraformaldehyde solution. A graded ethanol series (70% to 100%) was used to dehydrate the samples, they were then cleared with xylene and embedded in paraffin wax. Serial sections (5 μm thickness) were cut by LEICA RM2135 rotary microtome (Leica Microsystems GmbH, Simi Valley, CA, USA), and stained with hematoxylin and eosin (H and E). Tissue sections were observed under the bright field on a Zeiss Axio Imager microscope (Carl Zeiss Microscopy LLC, White Plains, NY, USA). 

### 2.7. Immunohistochemistry (IHC)

Immunohistochemical staining of jejunum sections was performed according to previous literature [20] to assess the levels of Nrf2 (Servicebio, Wuhan, China). Secondary antibodies were goat anti-rabbit IgG (Servicebio, Wuhan, China). In simple terms, paraffin sections were dewaxed into water and placed in citric acid antigen repair buffer for antigen repair. The slices were placed in 3% hydrogen peroxide solution and incubated in the dark for 25 min to block endogenous peroxidase. Then, 3% bovine serum albumin was added into the histochemical circle to seal it. After removing the sealing solution, the primary antibody was added for incubation overnight, and then the secondary antibody was added for incubation. Color solution was added to develop color. The nucleus was counterstained. Finally, the section was dehydrated and the tablet sealed. The sections were observed by Zeiss Axio Imager microscope (Carl Zeiss Microscopy LLC, White Plains, NY, USA). 

### 2.8. Statistics

The results were reported as the mean ± standard error of the mean (SEM). Data were analysed using one-way analysis of variance (ANOVA), followed by Tukey’s multiple-comparison (SPSS 20.0, IBM, Armonk, NY, USA). GraphPad Prism (version 8.0.2, San Diego, CA, USA) was used for the graphical representations. *p* < 0.05 indicates statistically significance.

## 3. Results

### 3.1. Therapeutic Ellagic Acid Treatment Reduced Diquat-Induced Weight Loss and Oxidative Damage

**Figure 1:**
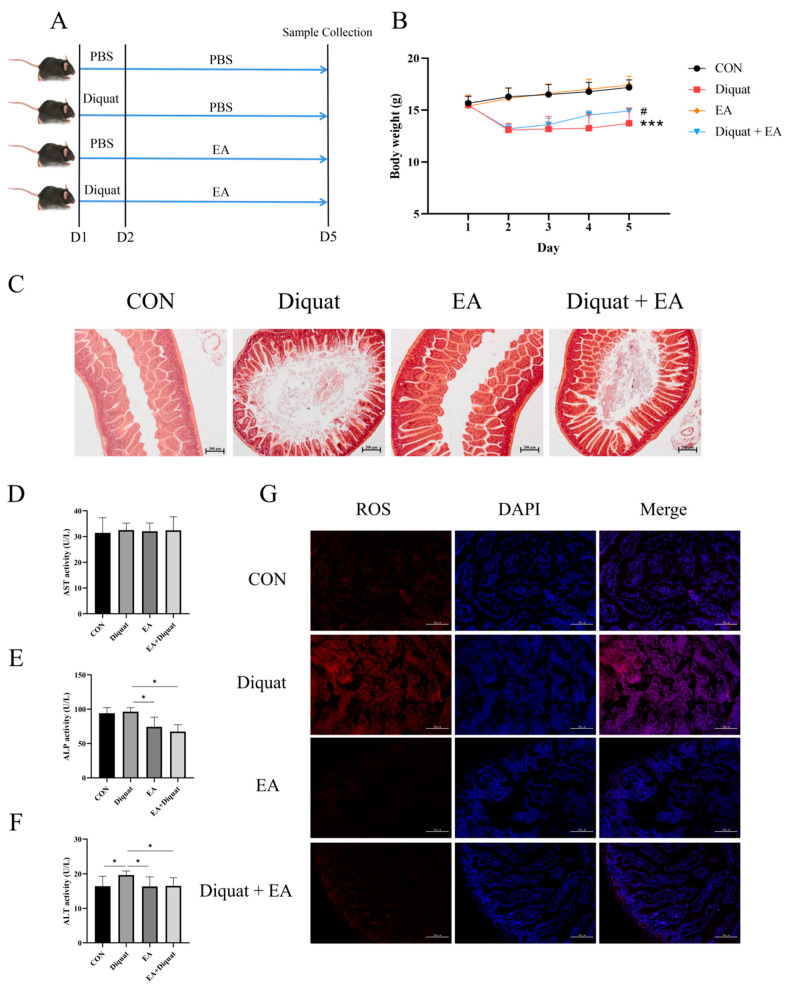
Effect of ellagic acid on weight loss and oxidative damage caused by diquat. (**A**) Experimental grouping scheme. (**B**) Body weight curve of mice. (**C**) HE staining of the jejunum. (**D**–**F**) The level of ALT, AST and ALP in serum. (**G**) ROS level in the jejunum. All data were presented as mean ± S.E.M (*n* = 4–6). *** means *p* < 0.001 when this group is compared with the CON group, # means *p* < 0.05 when this group is compared with the Diquat group, * means *p* < 0.05 between the two groups. PBS, phosphate buffer saline; CON, control; EA, ellagic acid; ALT, alanine aminotransferase; ALP, alkaline phosphatase; ROS, reactive oxygen species; DAPI, 4′,6-diamidino-2-phenylindole.

As shown in Figure 1B, mice stimulated with diquat had significantly lower body weight than the mice in the CON group. The Diquat + EA group had higher body weight than the Diquat group (*p* < 0.05), which indicated that EA alleviated the diquat induced body-weight loss to some extent. At the same time, EA treatment alleviated intestinal villi damage caused by diquat (Figure 1C) and reduced the ROS content in the jejunum (Figure 1G). The ALT activity (parameters of hepatoxicity) of the Diquat group on day 5 was the highest among the four groups (Figure 1F) (*p* < 0.05). Both the EA group and the EA + Diquat group lowered ALP and ALT activity when treated with 100 mg/kg EA for 5 days after the diquat injection (*p* < 0.05). Additionally, as shown in Figure S1, EA treatment shortened the time to return to initial body weight after diquat injection.

### 3.2. Ellagic Acid Upregulated Oxidative Stress Related Gene mRNA Expression in the Jejunum When Mice Challenged with Diquat

**Figure 2:**
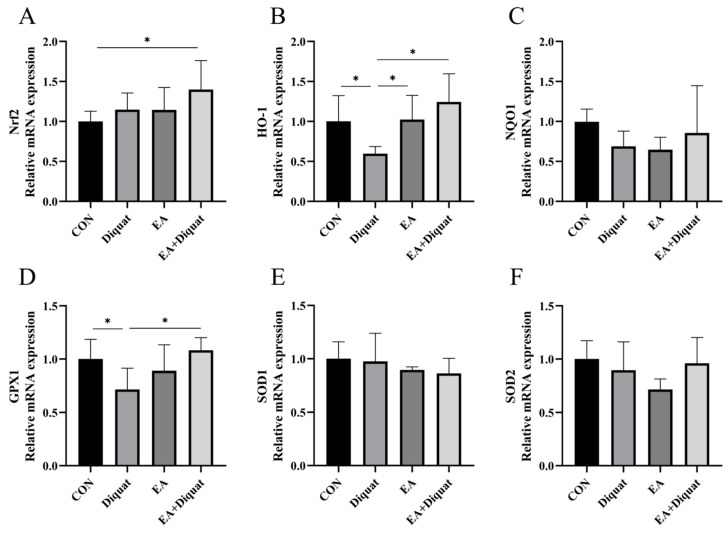
Effects of ellagic acid on antioxidant gene expression in the jejunum when mice were challenged with diquat. (**A**–**F**) The relative mRNA expression of Nrf2, HO-1, NQO1, GPX1, SOD1 and SOD2. All data were presented as mean ± S.E.M (*n* = 6). * means *p* < 0.05 between the two groups. CON, control; EA: ellagic acid; Nrf2, nuclear factor erythroid 2-related factor 2; HO-1, heme oxygenase-1; NQO-1, NADPH dehydrogenase-1; GPX1, glutathione peroxidase 1; SOD1, superoxide dismutase 1; SOD2, superoxide dismutase 2.

As shown in Figure 2, the Diquat group (25 mg/kg body weight diquat injection at day 1) had significantly decreased the GPX1 and HO-1 mRNA expression of the mice’s jejunum tissue at day 5 compared with the Control group and EA + Diquat group. Meaningfully, oral administration of 100 mg/kg EA for 5 days after diquat stimulation on day 1 significantly increased the Nrf2 mRNA expression of the mice’s jejunum tissue on day 5 compared with CON group (*p* < 0.5). However, NQO1, SOD1 and SOD2 mRNA expression in the jejunum had no significant difference among the four groups. 

### 3.3. Specific Nrf2 Inhibitor (ML385) Inhibited the Therapeutic Effects of Ellagic Acid on Weight Loss and Jejunum Oxidative Stress Caused by Diquat

**Figure 3:**
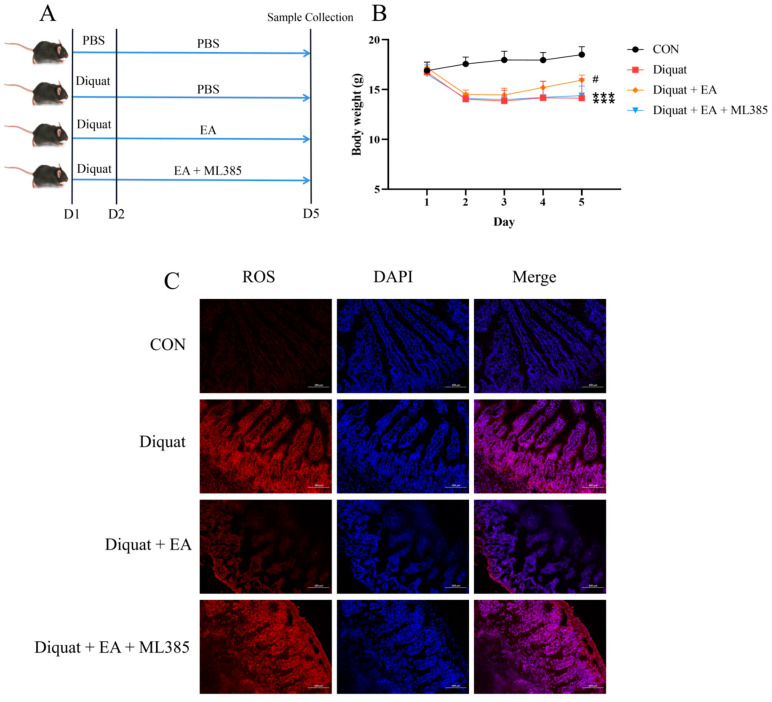
Specific Nrf2 inhibitor (ML385) inhibited the therapeutic effects of ellagic acid on weight loss and increased the ROS level in the jejunum caused by diquat. (**A**) Experimental grouping scheme. (**B**) Body weight curve of mice. (**C**) ROS level in the jejunum. All data were presented as mean ± S.E.M (*n* = 4–6). *** means *p* < 0.001 when this group compared with the CON group, # means *p* < 0.05 when this group compared with the Diquat group and the Diquat + EA + ML385 group. PBS, phosphate buffer saline; CON, control; EA, ellagic acid; ROS, reactive oxygen species; DAPI, 4′,6-diamidino-2-phenylindole.

To explore whether the therapeutic effects of ellagic acid on oxidative damage induced by diquat was in a Nrf2-dependent manner, we further applied specific Nrf2 inhibitor (ML385). As shown in Figure 3B, mice treated either with diquat or diquat + EA + ML385 had lower body weight on day 5 than that in the control group (*p* < 0.001). The Diquat + EA group had higher body weight than the Diquat + EA + ML385 group (*p* < 0.05), which demonstrated that the ML385 inhibited the improvement of EA on body weight loss caused by diquat. Similarly, the EA + Diquat group had lower ROS in the jejunum tissue compared with the Diquat group and the Diquat + EA + ML385 group (Figure 3C), which indicated that the ML385 inhibited the therapeutic effects of ellagic acid on jejunum oxidative damage caused by diquat.

### 3.4. Ellagic Acid Alleviated Diquat-Induced Jejunal Oxidative Stress through Nrf2 Mediated Signaling Pathway

**Figure 4:**
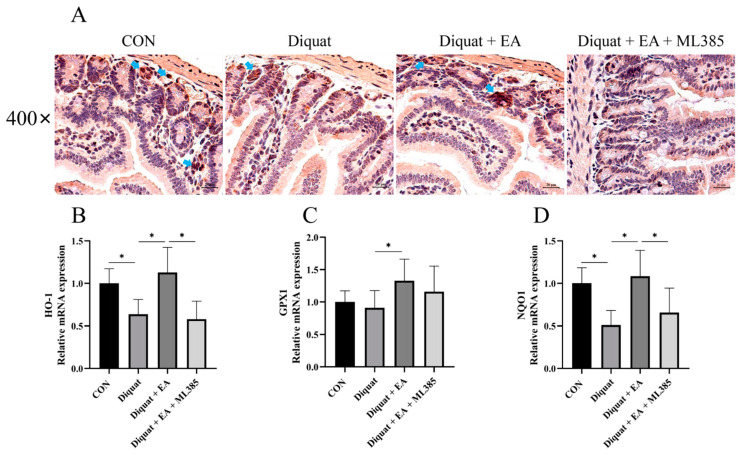
Ellagic acid alleviated diquat-induced jejunal oxidative stress through a Nrf2 mediated signaling pathway. (**A**) Immunohistochemistry analysis for Nrf2 in the jejunum tissue. (**B**–**D**) NQO-1, GPX1, and HO-1 mRNA expressions in jejunum tissue. All data were presented as mean ± S.E.M (*n* = 4–6). * means *p* < 0.05 between two groups. CON, control; EA, ellagic acid; Nrf2, nuclear factor erythroid 2-related factor 2; NQO-1, NADPH dehydrogenase-1; GPX1, glutathione peroxidase 1; HO-1, heme oxygenase-1.

Further, we examined Nrf2 expression with immunohistochemistry and related gene mRNA expression of the mice’s jejunum tissue on day 5. As shown in Figure 4, therapeutic treatment with EA (100 mg/kg BW/day) significantly increased Nrf2 immunostaining in the jejunum tissue after diquat injection in mice, while ML385 (specific Nrf2 inhibitor) inhibited the Nrf2 immunostaining as expected. The Diquat + EA group had significantly increased the NQO1, GPX1 and HO-1 mRNA expression of the mice’s jejunum tissue on day 5 compared with the Diquat group (*p* < 0.05), which was inhibited by the ML385 as shown by the gene expression in the Diquat + EA + ML385 group compared with the Diquat + EA group.

### 3.5. Prophylactic Ellagic Acid Treatment Ameliorated Diquat-Induced Jejunal Oxidative Damage

**Figure 5:**
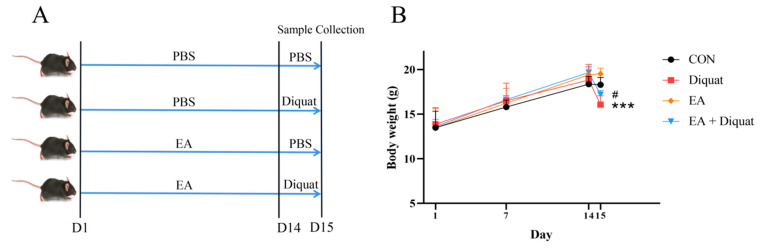

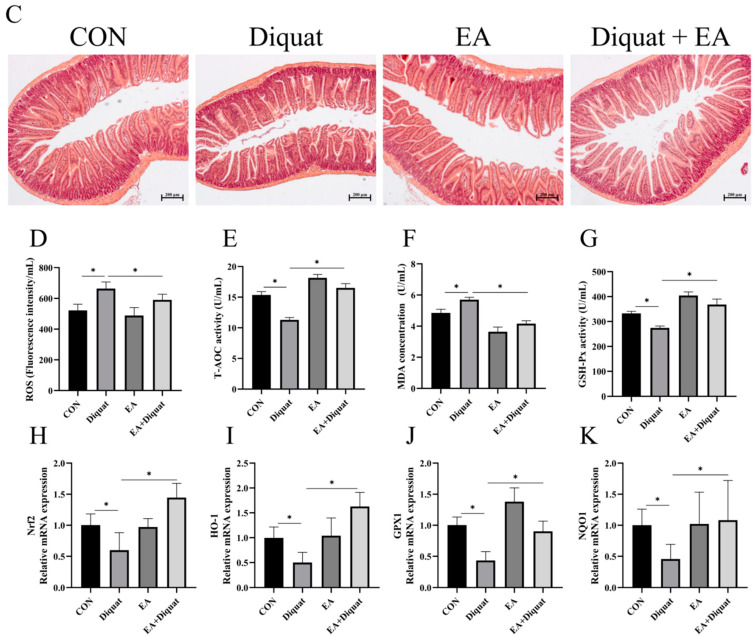
Effects of EA pre-treatment on oxidative stress induced by diquat. (**A**) Experimental grouping scheme. (**B**) Body weight curve of mice. (**C**) HE staining of the jejunum. (**D**) The fluorescence intensity of ROS in serum. (**E**) T-AOC in serum. (**F**) MDA concentration in serum. (**G**) GSH-Px activity. (**H–K**) The mRNA expression of Nrf2, HO-1, GPX1 and NQO1. All data were presented as mean ± S.E.M (*n* = 6). # means that this group had a significant difference compared with the Diquat group. *** means *p* < 0.001 when this group is compared with the CON group. * means *p* < 0.05 between the two groups. PBS, phosphate buffer saline; CON, control; EA, ellagic acid; ROS, reactive oxygen species; T-AOC, total antioxidant capacity; GSH-Px, glutathione peroxidase; MDA, malondialdehyde; Nrf2, nuclear factor erythroid 2-related factor 2; NQO-1, NADPH dehydrogenase-1; GPX1, glutathione peroxidase 1; HO-1, heme oxygenase-1.

Finally, we investigated the preventive effect of EA on diquat challenged mice. As shown in Figure 5B, only EA treatment did not change the body weight of mice, compared with the CON group. Diquat stimulation on day 15 obviously caused body weight loss of mice, and oral administration of EA for 14 days significantly alleviated body weight loss induced by diquat in mice (Figure 5B). EA treatment significantly increased T-AOC and GSH-Px activity, and reduced ROS and MDA levels in serum (Figure 5D–G). As shown in Figure 5H–K, diquat stimulation decreased mRNA expression of Nrf2, HO-1, NQO-1 and GPX1 in the jejunum (*p* < 0.05). However, EA pre-treatment to the diquat-challenged mice increased Nrf2, GPX1 and HO-1 gene expression (*p* < 0 05), potentially indicating that EA alleviated diquat induced oxidative stress by the Nrf2 mediated signaling pathway.

## 4. Discussion

Excessive production of ROS has many adverse effects on the body, such as intestinal damage, which further affect the body’s health [1,2,3]. Therefore, the elimination of excessive ROS is worth exploring. Ellagic acid is a natural polyphenol with strong scavenging ability of the oxygen free radicals and antioxidant activity [12]. Based on previous studies [21,22], this study used diquat to build an oxidative stress model and investigated the effects and mechanism of EA in alleviating oxidative stress and intestinal injury. The results demonstrated that EA supplementation can improve diquat-induced oxidative stress, and this effect is achieved through the Nrf2 mediated signaling pathway.

As the main organ for nutrient digestion and absorption, the intestine is particularly important for body health. The intestine is located at the junction of the body and the lumen environment, so it is more likely to receive oxidative damage that affects its normal physiological function [4,5,6]. Diquat, a strong oxidant, can induce ROS production via a redox circle [21,23]. Multiple studies have shown that diquat can induce intestinal oxidative stress in animals. This oxidative stress can not only cause intestinal damage, destroy the morphological structure of intestinal villi, but can also cause liver damage [7,24,25,26]. Our results replicated a similar phenomenon: diquat-induced oxidative stress significantly reduced the body weight of mice, produced a large number of ROS and damaged the morphological structure of the jejunum villi. Meanwhile, we found EA supplementation significantly improved diquat-induced weight loss, reduced the production of ROS in the intestine and improved intestinal villus morphology. This suggests that EA has a protective effect against jejunum damage caused by diquat. According to previous studies, EA can ameliorate rotenone-induced neurotoxicity in dopamine neurons and oxidative damage induced by high glucose in HepG2 cells by activating antioxidant pathways [14,27]. Therefore, we hypothesized that the improvement of EA in inducing oxidative stress in vivo is closely related to the activation of antioxidant pathways.

Oxidative stress, also known as an imbalance between antioxidant and oxidative systems, leads to ROS overload. Under physiological conditions, antioxidant enzymes such as GPX, HO-1 and SOD can remove excess oxygen free radicals and maintain oxidative balance [28,29,30,31,32]. Our results showed that diquat significantly reduced the mRNA expression of HO-1 and GPX1. HO-1 can catalyze the catabolism of heme and prevent it from promoting oxidation, while its byproducts have a slow reaction to remove ROS effectively [31]. GPX1 can reduce hydroperoxide production by using oxidized glutathione to prevent oxidative damage [32]. This suggests that diquat can disrupt the antioxidant system and induce accumulation of excessive ROS. At the same time, we found that EA supplementation before or after diquat stimulation could enhance antioxidant enzyme expression, such as HO-1 and GPX1 in the treatment trial and HO-1 and NQO1 in the prevention trial. These observations suggest that EA can respond to oxidative damage induced by diquat via enhancing the expression of antioxidant enzymes.

Nrf2 is a key sensor and transcription factor of the antioxidant system [9]. When ROS is overproduced in the body, Nrf2 in the cytoplasm moves into the nucleus and regulates the gene expression of downstream antioxidant enzymes such as HO-1, NQO1 and GPX1 [33]. Therefore, the expression levels of Nrf2 and its downstream genes were detected in this study. The results showed that EA supplementation significantly increased gene expression of Nrf2 and downstream antioxidant enzymes HO-1 and GPX1 regardless of supplementation before or after diquat stimulation. Interestingly, EA supplementation without diquat stimulation did not increase gene expression levels of Nrf2 and downstream antioxidant enzymes. A previous study showed similar results, suggesting that EA increased Nrf2, HO-1 and NQO1 levels only under rotenone stimulation [27]. We speculate that this may be because EA activates the antioxidant system only in response to oxidative stress. The results suggest that EA can alleviate or prevent diquat-induced oxidative stress by increasing the expression of antioxidant enzymes through activating the Nrf2 mediated signaling pathway. 

Subsequently, ML385, an inhibitor of Nrf2 protein, was used in the treatment to explore whether EA’s resistance to oxidative stress depended on the Nrf2 mediated signaling pathway. We found that the use of ML385 almost eliminated the palliative effect of EA. There was no difference in the jejunum ROS content and downstream antioxidant gene expression between the Diquat group and the Diquat + EA + ML385 group. Therefore, the improvement of EA to diquat-induced oxidative damage depends on the Nrf2 mediated signaling pathway.

## 5. Conclusions

In conclusion, orally-administered EA was effectively able to reduce diquat-induced weight loss and mitigate oxidative stress through promoting antioxidant capacity, which was achieved in an Nrf2-dependent manner.

## Figures and Tables

**Table 1:** Primer sequences used for quantitative RT-PCR.

Gene	Primer Sequences (5′-3′)	Primer Length (bp)	Product Length (bp)
β-actin F	GGCTGTATTCCCCTCCATCG	20	154
β-actin R	CCAGTTGGTAACAATGCCATGT	22	
HO-1 F	AAGCCGAGAATGCTGAGTTCA	21	100
HO-1 R	GCCGTGTAGATATGGTACAAGGA	23	
NQO1 F	AGGATGGGAGGTACTCGAATC	21	144
NQO1 R	AGGCGTCCTTCCTTATATGCTA	22	
SOD1 F	AACCAGTTGTGTTGTCAGGAC	21	139
SOD1 R	CCACCATGTTTCTTAGAGTGAGG	23	
SOD2 F	CAGACCTGCCTTACGACTATGG	22	113
SOD2 R	CTCGGTGGCGTTGAGATTGTT	21	
GPX1 F	CCACCGTGTATGCCTTCTCC	20	105
GPX1 R	AGAGAGACGCGACATTCTCAAT	22	
Nrf2 F	CTGAACTCCTGGACGGGACTA	21	182
Nrf2 R	CGGTGGGTCTCCGTAAATGG	20	
CAT F	GGAGGCGGGAACCCAATAG	19	102
CAT R	GTGTGCCATCTCGTCAGTGAA	21	

## Data Availability

Data are available from the corresponding author on reasonable request.

## Supplementary Materials

The following supporting information can be downloaded at: https://www.mdpi.com/article/10.3390/nu14051103/s1, Figure S1: Therapeutic ellagic acid treatment shortened the time for the mice to return to their initial weight when challenged with diquat.
