# Supplementary material for: Ellagic Acid Alleviates Diquat-Induced Jejunum Oxidative Stress in C57BL/6 Mice through Activating Nrf2 Mediated Signaling Pathway
Source: Nutrients. 2022 Mar 5;14(5):1103. doi: 10.3390/nu14051103 (PMC8912502; doi:10.3390/nu14051103)
Supplement: Supplementary file 1 [file nutrients-14-01103-s001.zip › nutrients-1575758-supplementary.pdf]

Supplementary Material

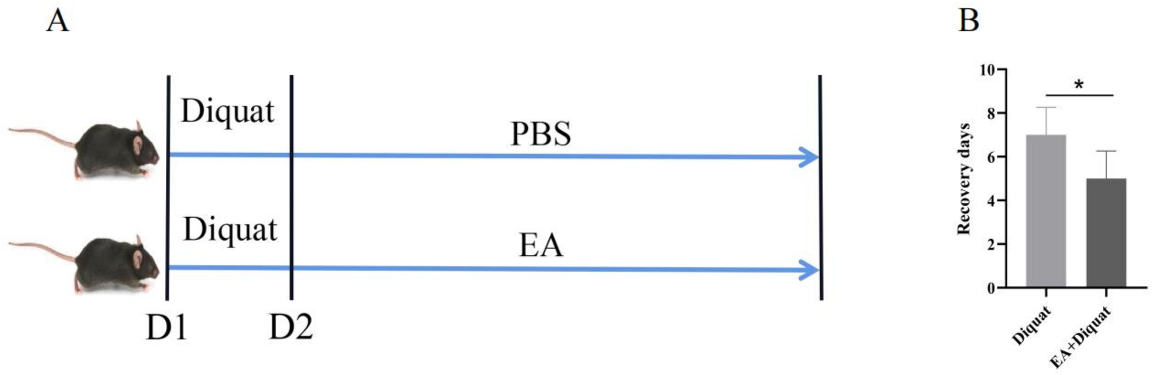

Figure S1: Therapeutic ellagic acid treatment shortened the time for the mice to return to their initial weight when challenged with diquat. (**A**) The treatment experimental scheme of mice. (**B**) The time for the mice to return to their initial weight when challenged with diquat. * means $p < 0.05$ beween two groups. All data were presented as mean ± S.E.M (n = 6).
